# Cross-cultural adaptation and validation of the Chinese version of the ankle joint functional assessment tool (AJFAT) questionnaire

**DOI:** 10.1186/s13047-023-00622-2

**Published:** 2023-04-26

**Authors:** Jinfeng Li, Fanji Qiu, Kirsten Legerlotz

**Affiliations:** 1 Department of Kinesiology, Iowa State University, Ames, IA 50011 USA; 2 Movement Biomechanics, Institute of Sport Sciences, Humboldt‐Universität zu Berlin, Unter Den Linden 6, 10099 Berlin, Germany

**Keywords:** Ankle sprains, CAI, Cumberland ankle instability tool, Cut off value, Reliability and validity evaluation

## Abstract

**Background:**

Ankle joint functional assessment tool (AJFAT) is gradually becoming a popular tool for diagnosing functional ankle instability (FAI). However, due to the lack of standard Chinese versions of AJFAT and reliability and validity tests, the use of AJFAT in the Chinese population is limited. This study aimed to translate and cross-culturally adapt the AJFAT from English into Chinese, and evaluate the reliability and validity of the Chinese version of AJFAT and to investigate its psychometric properties.

**Methods:**

The translation and cross-cultural adaptation of AJFAT was performed according to guidelines for cross-cultural adaptation of self-report measures. 126 participants with a history of ankle sprain completed the AJFAT-C twice within 14 days and completed the Cumberland ankle instability tool (CAIT-C) once. Test–retest reliability, internal consistency, ceiling and floor effects, convergent and structure validity and discriminative ability were investigated.

**Results:**

The test–retest reliability (ICC = 0.91, 95%CI = 0.87–0.94) and internal consistency (Cronbach’s alpha = 0.87) of the AJFAT-C were excellent. No ceiling or floor effects were detected. A moderate correlation between the AJFAT-C and the CAIT-C suggested a moderate convergent validity. The AJFAT-C had a two-factor structure: 1. function of the unstable side of the ankle joint (9 items) and 2. symptoms of the unstable side of the ankle (2 items). The ideal cut-off point of the AJFAT-C was calculated as 26 points.

**Conclusion:**

The Chinese version of AJFAT can be considered as a valid and reliable ankle joint function evaluation tool that can be applied in clinical and research work.

**Supplementary Information:**

The online version contains supplementary material available at 10.1186/s13047-023-00622-2.

## Introduction

Ankle sprains are a common musculoskeletal injury associated with physical activity [1–3]. In physically active populations, the cumulative incidence of ankle sprain was described to be as high as 7 per 1,000 units of exposure to court/indoor sports and 4.9 per 1,000 h of training [4]. There is also evidence that unstable foot and ankle joints are predisposed to develop degenerative arthritis and pain [5]. Ankle sprains lead to symptoms of pain, swelling, decreased muscle strength, and of 55%-72% might develop into chronic ankle instability (CAI) [6, 7]. A neuromuscular control deficit is considered a main cause resulting in CAI, and patients with CAI experience unstable feeling or "give way" in the ankle joint during daily and exercise activities [8]. Functional impairments are thus not necessarily accompanied by structural damage in the ankle joint as identified by medical imaging [8, 9].

From a kinematic perspective, CAI is characterized by joint movements that may not necessarily exceed normal physiological ranges but are still out of voluntary control [10]. Although there are many ways to assess the anatomical structure of the ankle joint in clinical practice, such as physical examination and imaging, there is no "gold standard" for the evaluation and diagnosis of CAI [8, 11, 12]. Various patient-reported outcome measures (PROMs) have been utilized to identify individuals with CAI [12–15]. Presently, the commonly used questionnaires for assessing chronic ankle instability include the Cumberland Ankle Instability Tool (CAIT) [13], the functional ankle instability questionnaire (FAIQ) [14], the identification of functional ankle instability (IdFAI) [12] and the ankle joint functional assessment tool (AJFAT) [15].

CAIT is widely used PROMs for patients with CAI [13, 16]. With only 9 items, it can save time during evaluation and reduce the burden on both patients and evaluators. The CAIT has been translated into several languages and has been proven to be an effective and reliable tool [13, 17–20]. It also has been translated into a Chinese version (CAIT-C) and was found to be good or excellent in test–retest reliability (ICC = 0.930), internal consistency (Cronbach’s alpha = 0.845–0.878) and responsiveness (ES = 1.316, SRM = 1.418) [21]. Unlike other CAI assessment tools. Unlike other CAI assessments where each ankle is compared to the contralateral side, CAIT conducts a separate assessment of each ankle's individual function. Therefore, patients with unilateral ankle instability need to spend additional time assessing healthy ankles. In contrast, AJFAT is designed to assess unilateral ankle instability, making it more suitable for patients with this condition. Additionally, AJFAT places greater emphasis on ankle stabilization during daily activities as compared to IdFAI.

Rozzi et al. compiled and developed the AJFAT in 1999 [13]. AJFAT contains 12 questions covering ankle pain, swelling, ability to walk on uneven ground, and ability to go up and down stairs. Reliability and validity have not been reported for this tool so far, and only one CAI-related study has tested the discriminative ability of AJFAT identifying a cut-off value of 26 points [22]. Like other CAI evaluation scales, AJFAT was written in English. If it is to be applied to different language and cultural environments, accurate translation and reliability evaluation are essential. To date, the AJFAT has not been translated into Chinese, with seems worthwhile given the large number of people who speak Chinese [23].

Thus, this study aims to translate the AJFAT into a Chinese version (AJFAT-C), and to evaluate the AJFAT-C in terms of test–retest reliability, internal consistency, ceiling and floor effects, validity and discriminative ability, with the aim of providing evidence to support the application of AJFAT-C in the screening and assessment process of CAI.

## Method

**Fig. 1:**
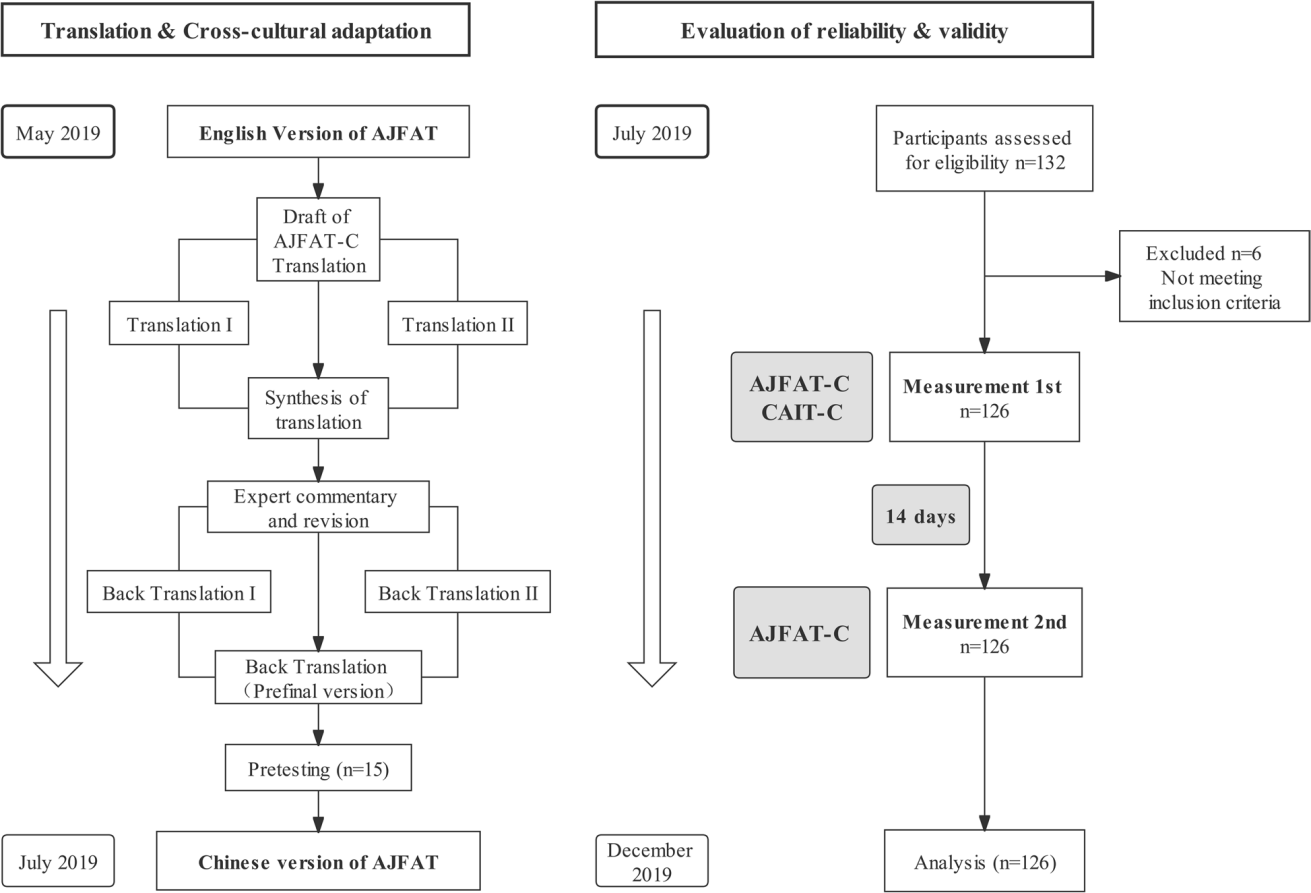
The flow chart of the cross-cultural adaptation and validation of AJFAT AJFAT-C: ankle joint functional assessment tool-Chinese. CAIT: Cumberland ankle instability tool

This study was approved by the Ethics Committee of Beijing Sport University (2019081H) and conducted in compliance with the 1964 declaration of Helsinki. Informed consent was obtained from patients before participating in the study. First the AJFAT was translated and cross-culturally adapted from English to Chinese; Second reliability and validity of the AJFAT-C were assessed (Fig. 1). We obtained authorization from the author of the original AJFAT.

### Instrument

The AJFAT evaluates ankle joint symptoms (pain, swelling), overall stability, strength and functional performance of the ankle joint. At the same time, the AJFAT requires the comparison between the affected side and the contralateral side, limiting the AJFAT to unilateral patients with CAI. The scoring standard is divided into 5 levels, corresponding to 0–4 points and resulting in a total score of the questionnaire of 48 points, with lower scores indicating impaired function. A score below 26 points is interpreted as CAI [22]. While the AJFAT-C was completed by the subjects, the scores remained hidden [15].

### Cross-cultural adaptation of AJFAT

The cross-cultural adaptation and translation of the AJFAT was carried out in accordance with the procedures of the cross-cultural adaptation of self-report measures [24]. Firstly, two translators who were proficient in English translated the questionnaire independently (a clinical rehabilitation therapist and a graduate student majoring in English) and compared the differences between the two translations. After thorough discussion a synthesis of the two translations was created. This common translation of the AJFAT-C was then evaluated and revised by two experts (a senior rehabilitation therapist and a sports medicine physician). The back translation process was carried out by two English-speaking translators (a Chinese student studying in Canada and a Chinese student studying in the United States) who translated the expert-revised AJFAT-C back into English. The back-translation was in agreement with the original version. To test this prefinal version, a pilot study was performed with 15 healthy college students who completed the AJFAT-C. Based on the feedback from these students, individual questions in the AJFAT-C were amended and the final version of AJFAT-C was generated (Table S1). The translation and adaptation process went smoothly and the structure of the original questionnaire remained unchanged. All of the 12 questions from the original AJFAT questionnaire were transferred to the AJFAT-C. The final version of AJFAT-C was developed by adjusting the order of words and repeatedly discussing certain words ("cut", "rolling", and "roll over") to ensure their appropriateness for the Chinese context. Additionally, translations were identified in conjunction with other assessment tools in the Chinese version.

### Subjects

A total of 126 college students filled out the paper version of the AJFAT-C from September 2019 to January 2020. The sample size was calculated based on the statistical guidelines that require a respondent-to-entry ratio of 10:1 [25]. All subjects were recruited by sending flyers in Beijing sport university. The inclusion criteria were as follow [26]: 1) over 18 years of age; 2) they reported at least once severe unilateral ankle sprains; 3) was associated with inflammatory symptoms; and 4) persistence of subjective ankle instability (giving way, and/or recurrent ankle sprain and /or feeling of instability). The exclusion criteria were as follow: 1) previous surgical history and/or fractures of the lower extremity; 2) other diseases like chronic inflammatory diseases that may impact ankle functions. Before the completion of the questionnaire, the investigators explained the meaning of questionnaire and collected basic information of subjects. Two weeks after the first completion of the AJFAT-C, the questionnaire was completed again by the same group of subjects to evaluate test–retest reliability. When the AJFAT-C was completed for the first time, the CAIT-C was completed as well to evaluate the validity of the AJFAT-C.

### Assessment of reliability and validity

The Chinese version of AJFAT was evaluated in five psychometric properties: test–retest reliability, internal consistency, ceiling and floor effects, validity and discriminative ability.

### Test–retest reliability

The intraclass correlation coefficient (ICC) was used to evaluate the AJFAT-C, with ICC > 0.9 indicating good reliability and ICC < 0.4 indicating poor reliability [27]. The standard error of the mean (SEM) was used to calculate the agreement between repeated measurements. The minimal detectable change (MDC) was also calculated. It reflects the minimum detectable within-person change of the score that can be interpreted as true change [28, 29]. SEM and MDC were calculated using the following Eqs.  [28]:

$$SEM\mathit=SD\times\sqrt{(1-ICC)}$$

$$MDC=1.96\times\sqrt2\times SEM$$

The systematic errors between two completions of the AJFAT-C were illustrated by plotting a Bland–Altman diagram (95% limits of agreement) [30].

### Internal consistency

The Cronbach's coefficient (Cronbach’s α) was used as an indicator to evaluate internal consistency [28]. A Cronbach's α value of > 0.70 was considered as strong, indicating high correlation between the questions and reflecting good internal consistency of the questionnaire [27, 31].

### Ceiling and floor effects

Ceiling and floor effects of the scale were defined as present if more than 15% of the subjects received the highest or lowest scores [28].

### Convergent validity

The convergent validity of AJFAT-C was examined by testing the correlation between the AJFAT-C and the CAIT-C, a validated questionnaire [23]. The CAIT is a widely used ankle instability scale [13, 16], and the Chinese version of this tool (CAIT-C) has been psychometrically tested, making it a relatively reliable assessment tool. All participants completed the CAIT-C at the initial measurement time point. The total CAIT-C score ranges from 0 to 30, with scores ≤ 27 identifying CAI [13, 32]. The Spearman's correlation coefficient was used to assess the relationship between CAIT-C and AJFAT-C, with correlation coefficients being judged as poor when < 0.30, moderate when between 0.30 and 0.50 and strong when > 0.50.

### Structural validity

Structural validity was tested by Principal Component Analysis (PCA) with varimax rotation to determine the dimensionality of the overall scale [33]. Only factors with eigenvalues ≥ 1 were considered. A prior measure of sample adequacy was conducted using Kaiser–Meyer–Olkin (KMO) and Bartlett's sphericity measure [25]. A KMO score below 0.5 is unacceptable, while scores between 0.50 and 0.60 are considered poor, scores between 0.60 and 0.70 are considered fair, scores between 0.70 and 0.80 are average, scores between 0.80 and 0.90 are good, and scores higher than 0.90 are very good. The associated significance for the Bartlett's sphericity test should be less than 0.001.

### Discriminative ability

Discriminative ability was evaluated to determine if the AJFAT-C can distinguish between young people with and without CAI. The Receiver Operating Characteristic (ROC) curve was plotted to confirm the cut-off point of the AJFAT-C using the Youden index [34]. The ideal cut-off point was determined using the maximum Youden index after calculating the specificity and diagnostic sensitivity for each potential cut-off score, with the formula [35]:

$$Youden\mathit=sensitivity\mathit+\mathit(specificity-1)$$

### Statistical analysis

According to the COSMIN checklist [29], in order to test the convergent validity, we need to firstly perform a hypothesis-driven correlation analysis before conducting the normal testing. The linear and normality hypotheses were tested by scatterplots, skewness, and ShapiroWilk's test. In regression analysis, the independence and normality of the residuals are tested using scatterplots. All statistical analyses were performed using IBM SPSS Statistics software for Windows, Version 21.0 (Chicago, Illinois, USA). Bland–Altman plots were drawn using MedCalc Statistical Software version 13.0.6 (Ostend, Belgium). Data were analysed for normality using the Shapiro–Wilks test. Continuous data are presented with a mean and standard deviation. An α level of 0.05 was considered as statistically significant.

## Results

In the first invitation, 132 participants completed AJFAT, but 6 participants (4.5%) were excluded as they did not meet the eligibility criteria. A total of 126 participants completed AJFAT-C twice. The basic information of subjects shows in Table 1.

**Table 1:** Demographic information of subjects

Characteristics	Number (%) or Mean ± SD
Age	22.2 ± 1.6
Sex	
Males	94 (74.6%)
Females	32 (25.4%)
Affected side	
Right	58 (46%)
Left	68 (54%)

*AJFAT-C* Ankle joint functional assessment tool-Chinese

### Test–retest reliability

**Fig. 2:**
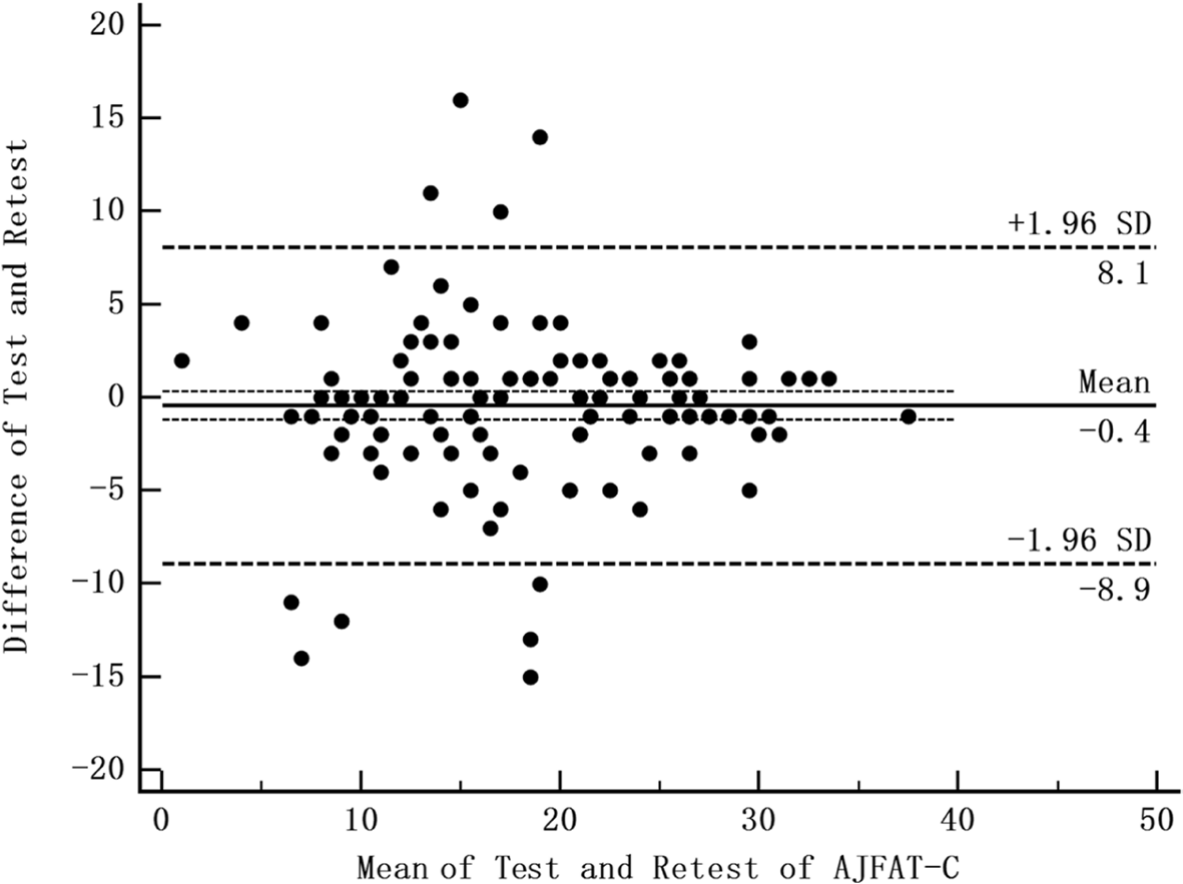
Bland–Altman plot with the test–retest results of 126 participants who completed the AJFAT-C twice. The horizontal dashed line represents the 95% (± 1.96 SD) agreement limit, the thin horizontal dashed line represents the 95% CI of the mean difference, and the horizontal solid line represents the mean of the difference. AJFAT-C: ankle joint functional assessment tool-Chinese

The test–retest reliability of the AJFAT-C was found to be excellent with an ICC of 0.91 (95% CI = 0.87–0.94). The SEM for all participants was 0.04 and the MDC was 0.12. The Bland–Altman plot (Fig. 2) showed a mean difference between AJFAT test and retest scores of -0.4 (95% limits of agreement -8.9 to 8.1).

### Internal consistency

The Cronbach’s α was 0.87, indicating strong internal consistency of the AJFAT-C. The Cronbach's α remained stable between 0.84 and 0.88 upon exclusion of question by question from analysis (Table 2).

**Table 2:** Internal consistency of the AJFAT-C

Questions	Cronbach’s α if item was deleted
Overall	0.867
Question 1	0.875
Question 2	0.866
Question 3	0.853
Question 4	0.845
Question 5	0.849
Question 6	0.849
Question 7	0.849
Question 8	0.842
Question 9	0.842
Question 10	0.871
Question 11	0.861
Question 12	0.880

*AJFAT-C* Ankle joint functional assessment tool-Chinese

### Ceiling and floor effects

There were no ceiling or floor effects detected for the AJFAT-C. Only one subject (0.8%) scored the minimum score of 0 points,

### Convergent validity

Totally, there were no missing items for CAIT-C and AJFAT-C. The scatterplots and Shapiro–Wilk test showed that the outcome variables of the two questionnaires were normally distributed. There were moderate and statistically significant correlations between the AJFAT-C and the CAIT-C (*r* = 0.39, *p* < 0.01). The results of the two questionnaires are shown in Table 3.

**Table 3:** Mean Scores per question for the AJFAT test and retest and the CAIT-C of the left and right ankle

Question number	AJFAT-C (Test)	AJFAT-C (Retest)	CAIT-C	
			Left ankle	Right ankle
Q1	1.47 ± 0.98^a^	1.53 ± 0.98	4.01 ± 1.37	4.10 ± 1.17
Q2	1.32 ± 0.89	1.46 ± 0.93	3.18 ± 0.76	3.20 ± 0.88
Q3	1.64 ± 0.97	1.64 ± 0.87	2.32 ± 0.75	2.38 ± 0.73
Q4	1.45 ± 0.94	1.48 ± 0.90	2.34 ± 0.92	2.40 ± 0.87
Q5	1.51 ± 0.98	1.53 ± 0.92	1.31 ± 0.65	1.32 ± 0.71
Q6	1.69 ± 0.79	1.61 ± 0.78	2.34 ± 0.71	2.36 ± 0.72
Q7	1.66 ± 0.75	1.60 ± 0.79	3.32 ± 0.78	3.26 ± 0.93
Q8	1.51 ± 0.96	1.44 ± 1.00	1.94 ± 1.05	1.89 ± 1.01
Q9	1.54 ± 0.87	1.55 ± 0.90	1.07 ± 1.26	1.26 ± 1.25
Q10	1.57 ± 1.04	1.72 ± 1.02	-	-
Q11	1.50 ± 1.04	1.67 ± 1.02	-	-
Q12	1.35 ± 1.57	1.47 ± 1.53	-	-
Total	18.22 ± 7.73	18.70 ± 7.59	21.83 ± 5.46	22.18 ± 5.42

^a^Data are presented as mean ± SD

*CAIT* Cumberland ankle instability tool

### Structural validity

The Kaiser–Meyer–Olkin measure verified the sampling adequacy for the analysis (KMO = 0.89). The Bartlett’s test of sphericity x^2^ = 1513.40, *p* < 0.001, indicated sufficiently large correlations between items to perform a principal component analysis.

**Fig. 3:**
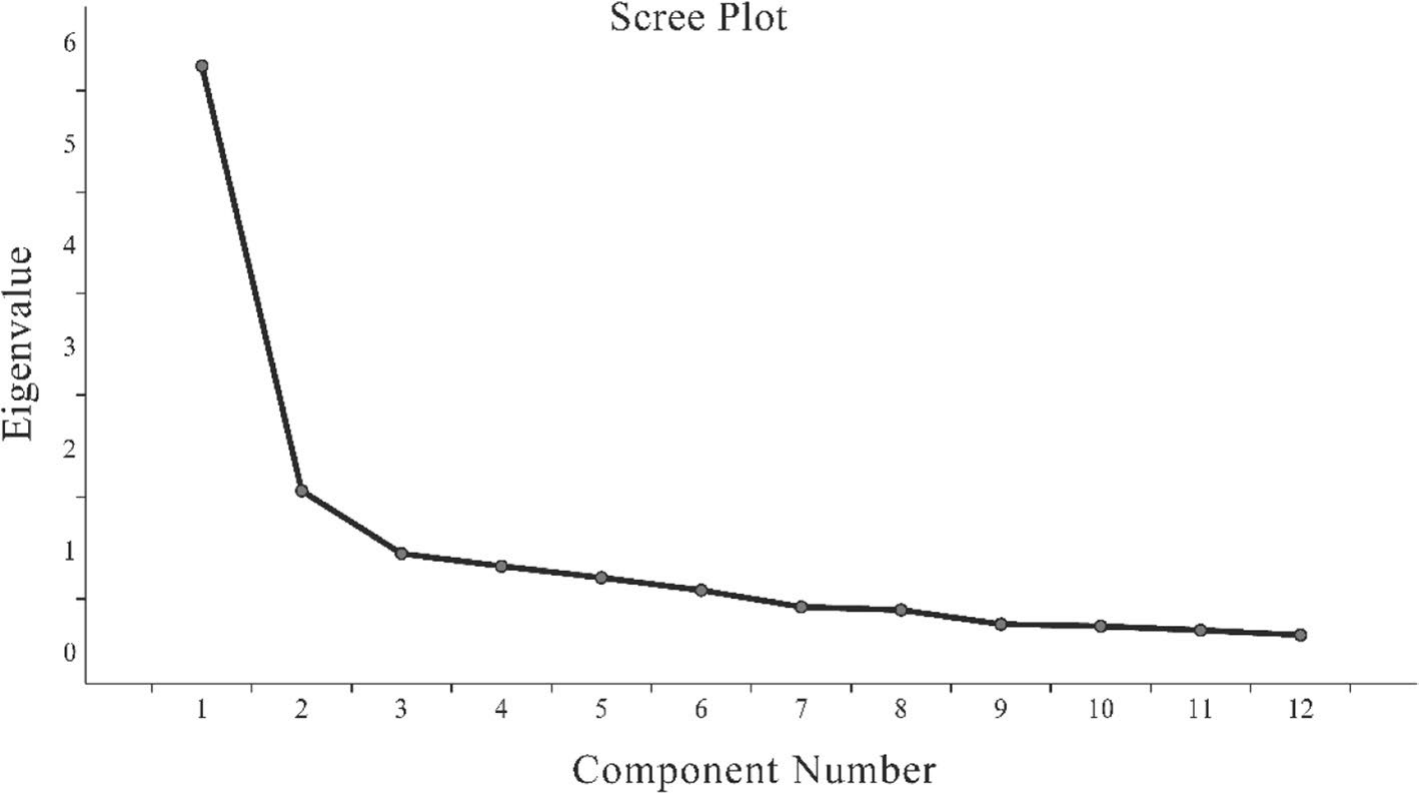
Scree plot of the AJFAT-C

Principal component analysis showed 2 underlying factors of the AJFAT-C (Table 4), with an explained variance of 47.8% and an eigenvalue of 5.7 and 13.1% explained variance and an eigenvalue of 1.6, respectively. Questions 3, 4, 5, 6, 7, 8, and 9 had the highest loadings (ranging from 0.77 to 0.87) on factor 1 (Table 4) which could be described as “Function of the unstable side of the ankle joint”. Questions 1 and 2 had the highest loadings (ranging from 0.85 to 0.87) on factor 2 which could be described as “Symptoms of the unstable side of the ankle” (Fig. 3).

**Table 4:** Principal components analysis of AJFAT-C

Components	Eigenvalue	Percentage of variance	Cumulative percentage of variance	Loading on factor 1	Loading on factor 2
1	5.74	47.84	47.84	-	0.85
2	1.56	13.03	60.87	-	0.87
3	0.95	7.88	68.75	0.77	-
4	0.82	6.86	75.61	0.84	-
5	0.71	5.91	81.52	0.81	-
6	0.59	4.88	86.39	0.84	-
7	0.42	3.52	89.91	0.80	-
8	0.39	3.26	93.17	0.87	-
9	0.25	2.09	95.26	0.83	-
10	0.23	1.94	97.20	-	-
11	0.19	1.60	98.80	-	-
12	0.14	1.20	100.00	-	-

### Discriminative ability

**Fig. 4:**
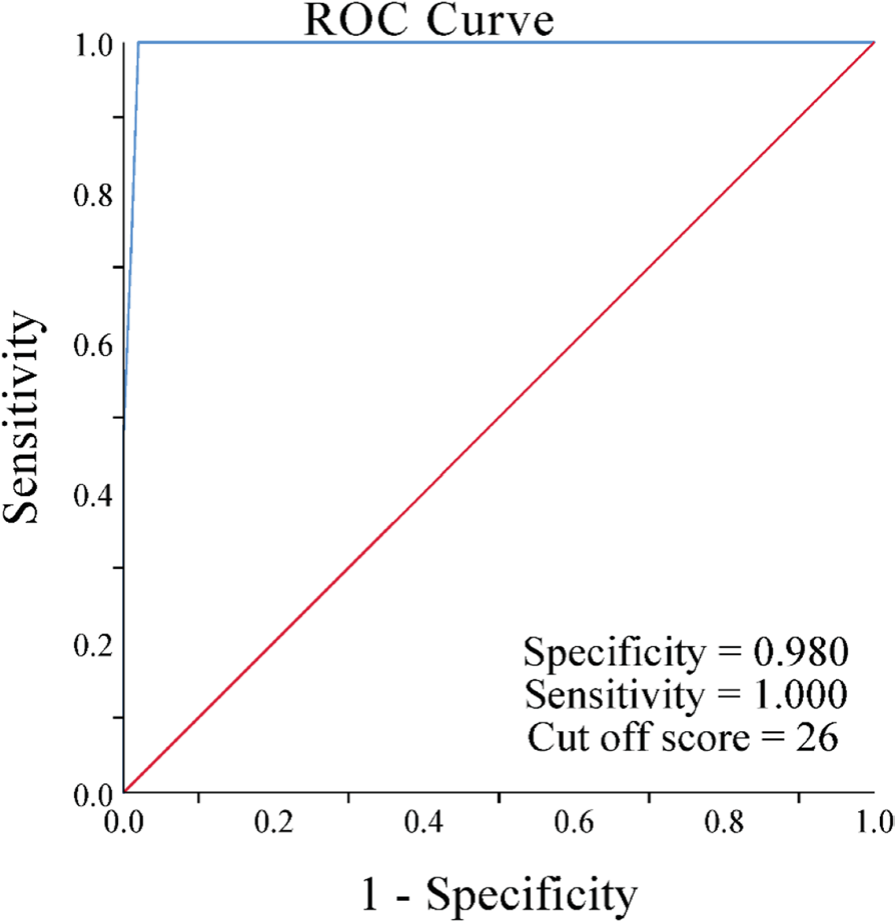
The Receiver Operating Characteristic (ROC) curve of the Ankle Function Assessment Tool. ROC is shown as a blue line. Reference line is shown as red line

The ROC curve of the AJFAT-C (Fig. 4) revealed an area under the curve (AUC) of 0.995 (*P* < 0.001). The maximum Youden index value was achieved at sensitivity = 1.00 and specificity = 0.98, indicating that an AJFAT-C total score ≥ 26 was the ideal cut-off point for distinguishing between CAI and non-CAI patients.

## Discussion

This is the first study to translate, adapt, and validate the English version of the AJFAT into Chinese for native Chinese speaking participants with CAI. This study provides evidence for the AJFAT-C measurement properties based on 126 young Chinese-speaking people. The AJFAT-C questionnaire showed high test–retest reliability and good internal consistency among questions; besides, the AJFAT-C had moderate construct validity using the CAIT-C as reference. There were no significant ceiling and floor effects.

Our findings suggest that the Chinese version of the AJFAT is a reliable and valid questionnaire for assessing CAI in young Chinese adults. As reliability of the AJFAT has not been reported yet, the AJFAT-C needs to be compared to other ankle function questionnaires in Chinese version which have been tested for reliability and validity. The ICC of the AJFAT-C was 0.91, which is comparable to other questionnaires that assess ankle instability, such as the CAIT-C [21], the Chinese IdFAI [23] and the Chinese FAAM [36], indicating that the AJFAT-C has a small measurement error. Moreover, the MDC value of the AJFAT-C was lower than that of the CAIT-C [21], suggesting that the AJFAT-C can detect small, meaningful within-person changes in a young population [28].

The high overall internal consistency of the AJFAT-C was similar to the Chinese FAAM [36] and the Chinese CAIT [21], and this result is also consistent with other CAI questionnaires in different languages [18, 20, 32]. The first question of the AJFAT-C showed the weakest correlation with the total score, possibly because it assessed "pain," while the other items evaluated ankle function. Although pain is a typical symptom of ankle instability [8], the degree of pain experienced by patients can vary widely, and severe functional impairment may not always coincide with severe pain.

Our study found that, similar to the CAIT-C [23], the AJFAT-C did not exhibit any ceiling or floor effects for the subjects, which means that none of the subjects had reached the lowest or highest points. This suggests that the AJFAT-C is capable of detecting varying levels of ankle instability and the results can accurately reflect the actual ankle joint function. Therefore, the AJFAT-C holds valuable reference value in the clinical diagnosis of CAI.

The moderate correlation in Spearman’s correlation coefficient between AJFAT-C and CAIT-C may be explained by the difference in the way the two questionnaires assessed CAI. The CAIT-C evaluated and recorded the scores of both ankles separately [23], while the AJFAT-C solely assessed and recorded the score of the unstable ankle [15]. Moreover, the variation in the number of questions asked could also contribute to the moderate correlation. In our study, principal component analysis revealed that nine questions of AJFAT-C were linked to two potential factors. As the original version of AJFAT has not undergone factor analysis and the two potential factors explained 60.869% of the variance in our study, we conclude that there is no need to remove any questions from AJFAT-C.

Identifying the cut-off points of the AJFAT-C can help increase the practical value of the tool. In this study, the AUC of AJFAT-C was 0.995, which was comparable to the value reported in a previous study by Ross et al. [22]. A cut-off score of ≥ 26 showed the highest sensitivity and specificity in distinguishing between CAI and Non-CAI patients, consistent with the findings of Ross et al. [22]. Our study and this previous research investigated similar age groups [22], suggesting that 26 points remain an optimal cut-off value for identifying the presence of CAI for Chinese-speaking young adults within our study's sample size.

Future research could explore additional psychometric properties of AJFAT, including conducting confirmatory factor analysis. The degree of convergence with other instruments with similar structures could also be examined. As the current validation of AJFAT was limited to participants with an average age of 22.2 years, it would be valuable to investigate its sensitivity to older adults with CAI.

## Limitation

The present study is not without limitations. First, we included only 126 participants, all of whom were young adults. Limitations in sample size and concentrated age intervals may have affected the reliability of the conclusions of this review. The AJFAT-C results are based on a comparison of the affected side with the contralateral limb. However, recent studies have shown that patients with unilateral ankle instability also depict some degree of ankle dysfunction in the other limb compared to healthy individuals [37–39], which could potentially be a limiting factor in the practical application of the AJFAT-C. Additionally, during the cross-cultural adaptation of the AJFAT-C, the healthy college students reported that some of the questions in the scale were not easy to answer for respondents without a history of ankle sprain. This highlights, that the practical application of the AJFAT-C is limited to patients with a history of ankle sprain and is not recommended for assessment ankle stability in healthy subjects. Furthermore, the responsiveness and sensitivity of AJFAT-C needs to be addressed in future studies performing clinical interventions with CAI patients. In addition, this study only calculated the MDC, future studies will need to further explore minimal clinical important difference (MICD) if the clinical significance of AJFAT is to be further explained. According to the COSMIN checklist, a pilot study should be performed in a patient population representing the target population (CAI patient), but in this study we only recruited healthy participants, which may result in the bias of the relevance of each item for the patients’ experience with the condition. Finally, self-reported questionnaires have many potential risks like lower response rate, due to lack of real-time feedback some subjects may not be able to answer questions accurately.

## Conclusion

This study found that AJFAT was easy to understand among CAI participants who spoke Chinese. The translated version contains terminology commonly used in Chinese and avoids overly technical terms. The Chinese version of AJFAT has shown good reliability and validity and can be considered as a reliable CAI evaluation tool. It can assist in the evaluation and detection of unilateral CAI in young Chinese-speaking adults in practice.

## Supplementary Information

**Additional file 1: Table S1.** AJFAT-C. **Appendix 1.** Ankle Joint Functional Assessment Tool. **Appendix 2.** Initial AJFAT-C. **Appendix 3.** Specific detail adjustments. **Appendix 4.** Results of ROC analysis.As per journal requirements, every additional file must have a corresponding caption. In this regard, please be informed that the caption was taken from the additional e-file itself. Please advise if the action taken is appropriate and amend if necessary. The action taken is appropriate.

## Data Availability

The data generated during and/or analysed during the current study are available from the corresponding author upon reasonable request.

## Abbreviations

AJFAT: Ankle joint functional assessment tool
AJFAT-C: The Chinese version of the ankle joint functional assessment tool
CAI: Chronic ankle instability
CAIT: Cumberland ankle instability tool
CAIT-C: The Chinese version of Cumberland ankle instability tool
Cronbach’s α: Cronbach's coefficient
ICC: Intraclass correlation coefficient
MDC: Minimal detectable change
PCA: Principal Component Analysis
ROC: Receiver Operating Characteristic
SEM: Standard error of the mean
