# Supplementary material for: Cross-cultural adaptation and validation of the Chinese version of the ankle joint functional assessment tool (AJFAT) questionnaire
Source: J Foot Ankle Res. 2023 Apr 26;16:22. doi: 10.1186/s13047-023-00622-2 (PMC10131472; doi:10.1186/s13047-023-00622-2)
Supplement: Supplementary file 1 — Additional file 1: Table S1. AJFAT-C. Appendix 1. Ankle Joint Functional Assessment Tool. Appendix 2. Initial AJFAT-C. Appendix 3. Specific detail adjustments. Appendix 4. Results of ROC analysis.As per journal requirements, every additional file must have a corresponding caption. In this regard, please be informed that the caption was taken from the additional e-file itself. Please advise if the action taken is appropriate and amend if necessary. The action taken is appropriate. [file 13047_2023_622_MOESM1_ESM.docx]

**Table S1: AJFAT-C**

| **1. 你如何描述受伤（不稳）侧踝关节的疼痛程度？**  ______(4) 比另一只脚踝轻很多  ______(3) 比另一只脚踝轻一点  ______(2) 和另一只脚踝相同  ______(1) 比另一只脚踝严重一点  ______(0) 比另一只脚踝严重很多  **2. 你如何描述受伤侧踝关节的肿胀程度？**  ______(4) 比另一只脚踝轻很多  ______(3) 比另一只脚踝轻一点  ______(2) 和另一只脚踝相同  ______(1) 比另一只脚踝严重一点  ______(0) 比另一只脚踝严重很多  **3. 你如何描述在不平坦表面上行走时受伤侧踝关节的能力？**  ______(0) 比另一只脚踝的能力差很多  ______(1) 比另一只脚踝的能力差一点  ______(2) 和另一只脚踝的能力相同  ______(3) 比另一只脚踝的能力好一点  ______(4) 比另一只脚踝的能力好很多  **4. 如何描述你对受伤侧踝关节稳定性的整体感觉？**  ______(0) 比另一只脚踝的稳定性差很多  ______(1) 比另一只脚踝的稳定性差一点  ______(2) 和另一只脚踝的稳定性相同  ______(3) 比另一只脚踝的稳定性好一点  ______(4) 比另一只脚踝的稳定性好很多  **5. 如何描述你对受伤侧踝关节力量的整体感觉？**  ______(0) 比另一只脚踝的力量弱很多  ______(1) 比另一只脚踝的力量弱一点  ______(2) 和另一只脚踝的力量相同  ______(3) 比另一只脚踝的力量强一点  ______(4) 比另一只脚踝的力量强很多  **6. 你如何评价下楼梯时受伤侧踝关节的稳定性？**  ______(0) 比另一只脚踝的稳定性差很多  ______(1) 比另一只脚踝的稳定性差一点  ______(2) 和另一只脚踝的稳定性相同  ______(3) 比另一只脚踝的稳定性好一点  ______(4) 比另一只脚踝的稳定性好很多  **7. 你如何评价慢跑时受伤侧踝关节的稳定性**  ______(0) 比另一只脚踝的稳定性差很多  ______(1) 比另一只脚踝的稳定性差一点  ______(2) 和另一只脚踝的稳定性相同  ______(3) 比另一只脚踝的稳定性好一点  ______(4) 比另一只脚踝的稳定性好很多  **8. 你如何评价跑步时受伤侧踝关节急停或变向的能力？**  ______(0) 比另一只脚踝的能力差很多  ______(1) 比另一只脚踝的能力差一点  ______(2) 和另一只脚踝的能力相同  ______(3) 比另一只脚踝的能力好一点  ______(4) 比另一只脚踝的能力好很多  **9. 你如何评价受伤侧踝关节的整体运动水平？**  ______(0) 比另一只脚踝的运动水平低很多  ______(1) 比另一只脚踝的运动水平低一点  ______(2) 和另一只脚踝的运动水平相同  ______(3) 比另一只脚踝的运动水平高一点  ______(4) 比另一只脚踝的运动水平高很多  **10. 下列哪项能最恰当地描述受伤侧踝关节感觉脚踝开始失稳（崴脚）的能力?**  ______(0) 比另一只脚踝的感觉慢很多  ______(1) 比另一只脚踝的感觉慢一点  ______(2) 和另一只脚踝的同时感觉  ______(3) 比另一只脚踝的感觉快一点  ______(4) 比另一只脚踝的感觉快很多  **11. 下列哪项能最恰当地描述当受伤侧踝关节在失稳（崴脚）发生时的反应能力?**  ______(0) 比另一只脚踝的反应慢很多  ______(1) 比另一只脚踝的反应慢一点  ______(2) 和另一只脚踝的同时反应  ______(3) 比另一只脚踝的反应快一点  ______(4) 比另一只脚踝的反应快很多  **12. 典型的脚踝失稳（崴脚）发生后，通常你会在多长时间内重返正常活动？**  ______(0) 超过2天  ______(1) 1至2天  ______(2) 一小时以上但是少于1天  ______(3) 15分钟至1小时  ______(4) 几乎是立刻 |
| --- |

**Appendix 1**

**Ankle Joint Functional Assessment Tool (AJFAT)**

| 1. How would you describe the level of pain you experience in your ankle?  ______(4) Much less than the other ankle  ______(3) Slightly less than the other ankle  ______(2) Equal in amount to the other ankle  ______(1) Slightly more than the other ankle  ______(0) Much more than the other ankle  2. How would you describe any swelling of your ankle?  ______(4) Much less than the other ankle  ______(3) Slightly less than the other ankle  ______(2) Equal in amount to the other ankle  ______(1) Slightly more than the other ankle  ______(0) Much more than the other ankle  3. How would you describe the ability of your ankle when walking on uneven surfaces?  ______(0) Much less than the other ankle  ______(1) Slightly less than the other ankle  ______(2) Equal in abilityt to the other ankle  ______(3) Slightly more than the other ankle  ______(4) Much more than the other ankle  4. How would you describe the overall feeling of stability od your ankle?  ______(0) Much less stable than the other ankle  ______(3) Slightly less stable than the other ankle  ______(2) Equal in stability to the other ankle  ______(1) Slightly more stable than the other ankle  ______(0) Much more stable than the other ankle  5. How would you describe the overall feeling of strength of your ankle?  ______(0) Much less strong than the other ankle  ______(3) Slightly less strong than the other ankle  ______(2) Equal in strength to the other ankle  ______(1) Slightly stronger than the other ankle  ______(0) Much stronger than the other ankle  6. How would you describe your ankle’s ability when you descend stairs?  ______(0) Much less than the other ankle  ______(1) Slightly less than the other ankle  ______(2) Equal in amount to the other ankle  ______(3) Slightly more than the other ankle  ______(4) Much more than the other ankle  7. How would you describe your ankle’s ability when you jog?  ______(0) Much less than the other ankle  ______(1) Slightly less than the other ankle  ______(2) Equal in amount to the other ankle  ______(3) Slightly more than the other ankle  ______(4) Much more than the other ankle  8. How would you describe your ankle’s ability to “cut” or change direction, when running?  ______(0) Much less than the other ankle  ______(1) Slightly less than the other ankle  ______(2) Equal in amount to the other ankle  ______(3) Slightly more than the other ankle  ______(4) Much more than the other ankle  9. How would you describe the overall activity level of your ankle?  ______(0) Much less than the other ankle  ______(1) Slightly less than the other ankle  ______(2) Equal in amount to the other ankle  ______(3) Slightly more than the other ankle  ______(4) Much more than the other ankle  10. Which statement best describes your ability to sense your ankle beginning to “roll over”?  ______(0) Much later than the other ankle  ______(1) Slightly later than the other ankle  ______(2) At the same time as the other ankle  ______(3) Slightly sooner than the other ankle  ______(4) Much sooner than the other ankle  11. Compared with the other ankle, which statement best describes your ability to respond to your ankle beginning to “roll over”?  ______(0) Much later than the other ankle  ______(1) Slightly later than the other ankle  ______(2) At the same time as the other ankle  ______(3) Slightly sooner than the other ankle  ______(4) Much sooner than the other ankle  12. Following a typical incident of your ankle “rolling”, which statement best describes the time required to return to activity?  ______(0) More than 2 days  ______(1) 1 to 2 days  ______(2) More than 1 hour and less than 1 day  ______(3) 15minutes to 1 hour  ______(4) Almost immediately |
| --- |

**Appendix 2**

**Initial AJFAT-C**

**1. 你如何描述受伤侧踝关节的疼痛程度？**

______(4) 比另一只脚踝轻很多

______(3) 比另一只脚踝轻一点

______(2) 和另一只脚踝相同

______(1) 比另一只脚踝严重一点

______(0) 比另一只脚踝严重很多

**2. 你如何描述受伤侧踝关节的肿胀程度？**

______(4) 比另一只脚踝轻很多

______(3) 比另一只脚踝轻一点

______(2) 和另一只脚踝相同

______(1) 比另一只脚踝严重一点

______(0) 比另一只脚踝严重很多

**3. 你如何描述在非平面上行走时受伤侧踝关节的能力？**

______(0) 比另一只脚踝的能力差很多

______(1) 比另一只脚踝的能力差一点

______(2) 和另一只脚踝的能力相同

______(3) 比另一只脚踝的能力好一点

______(4) 比另一只脚踝的能力好很多

**4. 如何描述你对受伤侧踝关节的整体稳定性？**

______(0) 比另一只脚踝的稳定性差很多

______(1) 比另一只脚踝的稳定性差一点

______(2) 和另一只脚踝的稳定性相同

______(3) 比另一只脚踝的稳定性好一点

______(4) 比另一只脚踝的稳定性好很多

**5. 如何描述你对受伤侧踝关节力量的整体感觉？**

______(0) 比另一只脚踝的力量弱很多

______(1) 比另一只脚踝的力量弱一点

______(2) 和另一只脚踝的力量相同

______(3) 比另一只脚踝的力量强一点

______(4) 比另一只脚踝的力量强很多

**6. 你如何评价下楼梯时受伤侧踝关节的稳定性？**

______(0) 比另一只脚踝的稳定性差很多

______(1) 比另一只脚踝的稳定性差一点

______(2) 和另一只脚踝的稳定性相同

______(3) 比另一只脚踝的稳定性好一点

______(4) 比另一只脚踝的稳定性好很多

**7. 你如何评价慢跑时受伤侧踝关节的稳定性**

______(0) 比另一只脚踝的稳定性差很多

______(1) 比另一只脚踝的稳定性差一点

______(2) 和另一只脚踝的稳定性相同

______(3) 比另一只脚踝的稳定性好一点

______(4) 比另一只脚踝的稳定性好很多

**8. 你如何评价跑步时受伤侧踝关节急停或变向的能力？**

______(0) 比另一只脚踝的能力差很多

______(1) 比另一只脚踝的能力差一点

______(2) 和另一只脚踝的能力相同

______(3) 比另一只脚踝的能力好一点

______(4) 比另一只脚踝的能力好很多

**9. 你如何评价受伤侧踝关节的整体运动水平？**

______(0) 比另一只脚踝的运动水平低很多

______(1) 比另一只脚踝的运动水平低一点

______(2) 和另一只脚踝的运动水平相同

______(3) 比另一只脚踝的运动水平高一点

______(4) 比另一只脚踝的运动水平高很多

**10. 下列哪项能最恰当地描述受伤侧踝关节感觉脚踝开始失稳的能力?**

______(0) 比另一只脚踝的感觉慢很多

______(1) 比另一只脚踝的感觉慢一点

______(2) 和另一只脚踝的同时感觉

______(3) 比另一只脚踝的感觉快一点

______(4) 比另一只脚踝的感觉快很多

**11. 下列哪项能最恰当地描述当受伤侧踝关节在失稳发生时的反应能力?**

______(0) 比另一只脚踝的反应慢很多

______(1) 比另一只脚踝的反应慢一点

______(2) 和另一只脚踝的同时反应

______(3) 比另一只脚踝的反应快一点

______(4) 比另一只脚踝的反应快很多

**12. 典型的脚踝失稳发生后，通常你会在多长时间内重返正常活动？**

______(0) 超过2天

______(1) 1至2天

______(2) 一小时以上但是少于1天

______(3) 15分钟至1小时

______(4) 几乎是立刻

**Appendix 3**

**Specific detail adjustments**

1. In the question 1, the English version only uses 'your ankle'. After translating into Chinese, participants did not understand that this refers to the ankle on the unstable side, so when modifying, we specifically refer the ankle to the ankle on the injured side.
2. In the question 3, the English version uses 'uneven surface'. In the first translation, we chose a vague Chinese word, so when revising it, we changed the word to ‘不平坦的表面’.
3. In questions 11 and 12, the original Chinese version only used '踝关节失稳', given that the term was too specialized, so many subjects asked the assessor what the question meant. In the second version we added a more colloquial '崴脚'.

**Appendix 4**

Results of ROC analysis

| Cut-off score | sensitivity | 1 - specificity | |
| --- | --- | --- | --- |
| -1.0000 | 1.000 | 1.000 |  |
| .5000 | 1.000 | .990 |  |
| 1.5000 | 1.000 | .980 |  |
| 2.5000 | 1.000 | .970 |  |
| 4.5000 | 1.000 | .960 |  |
| 6.5000 | 1.000 | .941 |  |
| 7.5000 | 1.000 | .921 |  |
| 8.5000 | 1.000 | .901 |  |
| 9.5000 | 1.000 | .842 |  |
| 10.5000 | 1.000 | .782 |  |
| 11.5000 | 1.000 | .733 |  |
| 12.5000 | 1.000 | .703 |  |
| 13.5000 | 1.000 | .634 |  |
| 14.5000 | 1.000 | .604 |  |
| 15.5000 | 1.000 | .505 |  |
| 16.5000 | 1.000 | .455 |  |
| 17.5000 | 1.000 | .436 |  |
| 18.5000 | 1.000 | .386 |  |
| 19.5000 | 1.000 | .327 |  |
| 20.5000 | 1.000 | .287 |  |
| 21.5000 | 1.000 | .208 |  |
| 22.5000 | 1.000 | .149 |  |
| 23.5000 | 1.000 | .089 |  |
| 24.5000 | 1.000 | .050 |  |
| 25.5000 | 1.000 | .020 |  |
| 26.5000 | .720 | .010 |  |
| 27.5000 | .480 | .000 |  |
| 28.5000 | .440 | .000 |  |
| 29.5000 | .360 | .000 |  |
| 30.5000 | .240 | .000 |  |
| 31.5000 | .200 | .000 |  |
| 32.5000 | .160 | .000 |  |
| 33.5000 | .080 | .000 |  |
| 35.5000 | .040 | .000 |  |
| 38.0000 | .000 | .000 |  |
